# A review of machine learning methods for imbalanced data challenges in chemistry

**DOI:** 10.1039/d5sc00270b

**Published:** 2025-04-22

**Authors:** Jian Jiang, Chunhuan Zhang, Lu Ke, Nicole Hayes, Yueying Zhu, Huahai Qiu, Bengong Zhang, Tianshou Zhou, Guo-Wei Wei

**Affiliations:** a Research Center of Nonlinear Science, School of Mathematical and Physical Sciences, Wuhan Textile University Wuhan 430200 P R. China jjiang@wtu.edu.cn; b Department of Mathematics, Michigan State University East Lansing Michigan 48824 USA; c Key Laboratory of Computational Mathematics, Guangdong Province, School of Mathematics, Sun Yat-sen University Guangzhou 510006 P R. China; d Department of Electrical and Computer Engineering, Michigan State University East Lansing Michigan 48824 USA; e Department of Biochemistry and Molecular Biology, Michigan State University East Lansing Michigan 48824 USA weig@msu.edu

## Abstract

Imbalanced data, where certain classes are significantly underrepresented in a dataset, is a widespread machine learning (ML) challenge across various fields of chemistry, yet it remains inadequately addressed. This data imbalance can lead to biased ML or deep learning (DL) models, which fail to accurately predict the underrepresented classes, thus limiting the robustness and applicability of these models. With the rapid advancement of ML and DL algorithms, several promising solutions to this issue have emerged, prompting the need for a comprehensive review of current methodologies. In this review, we examine the prominent ML approaches used to tackle the imbalanced data challenge in different areas of chemistry, including resampling techniques, data augmentation techniques, algorithmic approaches, and feature engineering strategies. Each of these methods is evaluated in the context of its application across various aspects of chemistry, such as drug discovery, materials science, cheminformatics, and catalysis. We also explore future directions for overcoming the imbalanced data challenge and emphasize data augmentation *via* physical models, large language models (LLMs), and advanced mathematics. The benefit of balanced data in new material design and production and the persistent challenges are discussed. Overall, this review aims to elucidate the prevalent ML techniques applied to mitigate the impacts of imbalanced data within the field of chemistry and offer insights into future directions for research and application.

## Abstract 2

Imbalanced data, where certain classes are significantly underrepresented in a dataset, is a widespread machine learning (ML) challenge across various fields of chemistry, yet it remains inadequately addressed.

## Graphical abstract

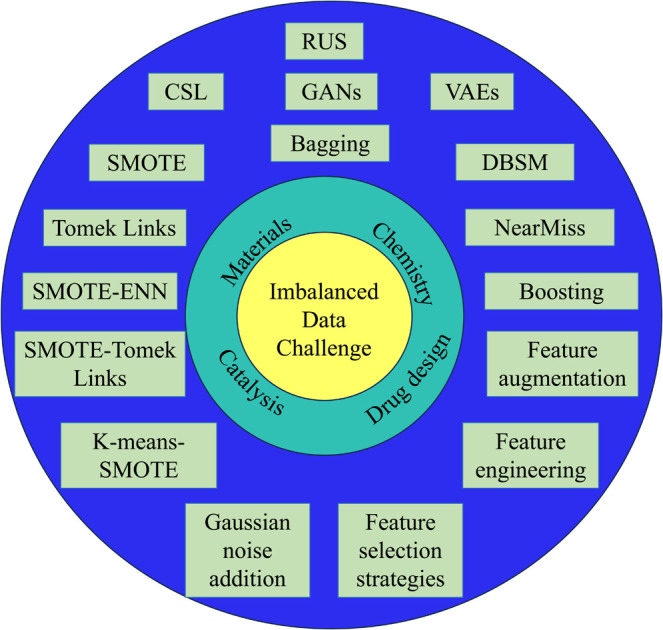

## 1 Introduction

The awarding of the 2024 Nobel Prize in Chemistry to David Baker for computational protein design and to Demis Hassabis and John M. Jumper for protein structure prediction underscores the growing influence of artificial intelligence (AI) in scientific discovery. As AI and machine learning (ML) become integral to advancing chemical research,^1^ one of the most pressing challenges is the issue of imbalanced data. In many chemical datasets, the disproportionate distribution of classes poses significant obstacles to the development of reliable and accurate models, particularly when applied to complex chemical phenomena.

Imbalanced data, a common phenomenon in data science, refers to significant disparities in the number of samples from different categories in classification tasks. The emergence of imbalanced data in chemistry is primarily attributed to the complexity and diversity of molecular data due to several factors. Naturally occurring biases in molecular distributions, where certain structures are more abundant than others, lead to a skew in data availability. Additionally, “selection bias” in sample collection processes can further exacerbate the imbalance. For instance, datasets may over-represent specific types of molecules or reactions due to experimental priorities or technical limitations. In drug discovery,^2^ active drug molecules are often significantly outnumbered by inactive ones due to the constraints of cost, safety, and time. Similarly, in molecular property prediction,^3^ models designed to assess toxicity often predict toxic outcomes more frequently, as toxic substances comprise a significant portion of the data. The study of protein–protein interactions also suffers from this imbalance, with experimentally validated interactions being much rarer than non-interactions.^4^

The presence of imbalanced data has direct implications for the performance of ML models. Most algorithms, such as random forests (RF) and support vector machines (SVM),^5^ assume a uniform distribution of data across categories.^6^ When trained on imbalanced datasets, models tend to focus on classes with more abundant data, often neglecting the minority classes. This bias results in models that are less sensitive to underrepresented features, which can critically undermine the accuracy of predictions in real-world applications. Consequently, overcoming the limitations imposed by imbalanced data is essential for the advancement of ML in chemical research.

Addressing the issue of imbalanced data in chemistry has become a major area of interest for researchers. Various strategies have been proposed, including resampling techniques like oversampling and undersampling, data augmentation, and ensemble algorithms. Feature engineering and selection methods have also been explored to mitigate the negative effects of data imbalance. However, despite the increasing body of work in this area, the existing reviews provide a general overview of these methods without specifically addressing their applications within chemistry. This review aims to fill this gap by offering a comprehensive overview of the imbalanced data challenge and solutions in chemical research, with a particular focus on recent advancements and their practical implications. Through this examination, we seek to provide researchers with a deeper understanding of the challenge posed by imbalanced data and to stimulate further progress in developing effective solutions.

The rest of this article is organized as follows. Sections 2.1–2.4 provide a detailed description of current technologies and algorithms for handling imbalanced data and demonstrate their applications in distinct fields of chemistry. Section 2.5 lists some indicators for evaluating model performance. In Section 3, we discuss new trends and challenges in the study of imbalanced data in chemistry and highlight future perspectives.

## 2 Current approaches and techniques

### 2.1 Resampling techniques

#### 2.1.1 Oversampling techniques

**Fig. 1:**
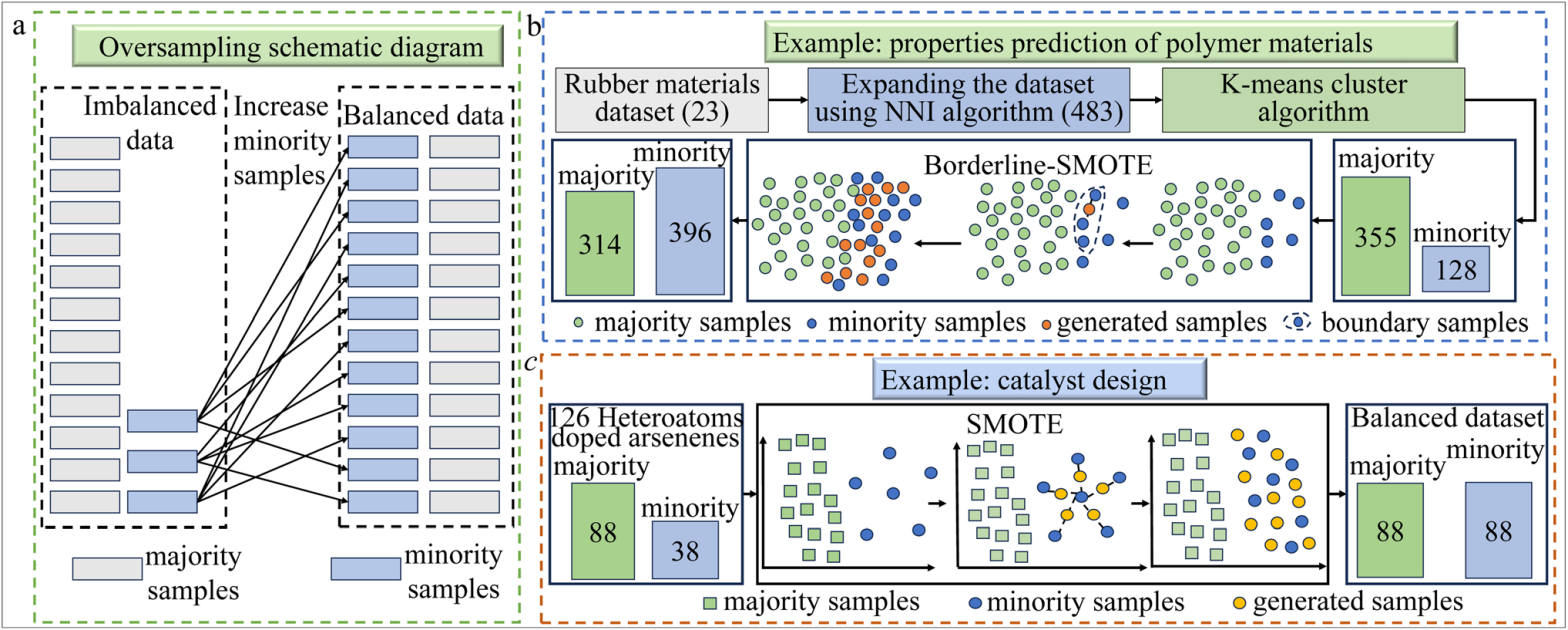
(a) A schematic diagram of an oversampling method, demonstrating the approach of the oversampling technique to balance the dataset. (b) This example demonstrates the application of Borderline-SMOTE method in properties prediction of polymer materials.^7^ Firstly, experimental data of 23 rubber materials were collected, and the nearest neighbor interpolation (NNI) algorithm was used to expand the dataset, resulting in a total of 483 datasets. Then, the K-means algorithm was used to cluster these datasets into two categories. Finally, based on the clustering results, Borderline-SMOTE was used to interpolate along the boundaries of the minority samples, generating two clusters with sample sizes of 314 and 396, respectively. (c) This illustration showcases the utilization of the SMOTE technique in the domain of catalyst development.^8^ 126 heteroatoms doped arsenenes were collected as the original dataset, and the absolute value of Gibbs free energy changes (|Δ*G*_H_|) of 0.2 eV was selected as the threshold to divide the original data into two categories (88 with |Δ*G*_H_| > 0.2 eV and 38 with |Δ*G*_H_| < 0.2 eV). Then, SMOTE was applied to solve the problem of data imbalance and obtain two types of evenly distributed data.

Oversampling is a widely used technique for addressing data imbalance, particularly when the minority class has significantly fewer samples than the majority class. By duplicating or generating new samples for the minority class while maintaining the original data distribution, oversampling helps balance class proportions. An example schematic diagram for oversampling is shown in Fig. 1a. This approach enhances the model's ability to learn the characteristics of the minority class, improving its predictive performance and reducing bias due to class imbalance. It is commonly applied in various fields of chemistry, such as genomics and transcriptomics,^9,10^ as well as drug design,^11,12^ quantum computing^13^ and materials design.^7^

One of the most prominent oversampling methods is the Synthetic Minority Over-sampling Technique (SMOTE), first introduced by Chawla *et al.* in 2002.^14^ SMOTE generates new minority class samples by synthesizing them from the existing data, which helps preserve the original feature distribution and mitigates overfitting. Its ability to enhance model generalization has led to widespread adoption across various chemistry domains. For instance, in materials design, SMOTE has been used to resolve class imbalance when integrated with Extreme Gradient Boosting (XGBoost) and nearest neighbor interpolation, improving the prediction of mechanical properties of polymer materials.^7^ The illustration of this process of balancing data is shown in Fig. 1b. Similarly, as part of an ML method trained on molecular dynamics data to predict the tensile stress of natural rubber, SMOTE is used to interpolate at a few sample boundaries to solve the problem of sample imbalance.^15^ In catalyst design, the authors used SMOTE to solve the problem of uneven data distribution in the original dataset, improving the predictive performance of ML models and promoting candidate screening of hydrogen evolution reaction catalysts.^8^ The illustration of SMOTE for balancing data for this catalyst design example is displayed in Fig. 1c.

However, SMOTE has limitations, such as introducing noisy data, struggling with complex decision boundaries, failing to account for internal distribution differences within the minority class, and requiring high computational costs. To address these issues, advanced oversampling techniques have been developed, including Borderline-SMOTE,^16^ SVM-SMOTE,^17^ RF-SMOTE,^18^ Safe-level-SMOTE,^19^ SMOTE-NC,^20^ and ADASYN.^21^ These methods refine SMOTE's approach by better handling class overlap, decision boundary complexity, and minority class distribution, expanding its applicability to more complex datasets. In drug discovery, the uneven distribution of active and inactive compounds affects the prediction accuracy of ML models. Therefore, in a search for new histone deacetylase 8 (HDAC8) inhibitors, Nurani *et al.* used SMOTE to construct a balanced dataset.^22^ They further selected an RF model, which demonstrated the best predictive performance on their training set compared to other tested ML methods, and the resulting RF-SMOTE prediction model was indicated to be helpful in identifying new HDAC8 inhibitors. In protein engineering, sample imbalance is a major challenge for predicting protein–protein interaction sites. As traditional SMOTE methods focus equally on every minority class sample, while Borderline-SMOTE methods are more sensitive to boundary samples, Jiang *et al.* used a CNN model with Borderline-SMOTE to predict protein–protein interaction sites, which is helpful for protein design and mutation analysis.^23^ Furthermore, a combination of the most distant undersampling and Safe-level-SMOTE oversampling techniques has been used to address data imbalance issues, demonstrating its excellent performance in balancing the number of lysine formylation sites and non-formylation sites and enabling the prediction of lysine formylation sites when paired with ML.^24^

#### 2.1.2 Undersampling techniques

**Fig. 2:**
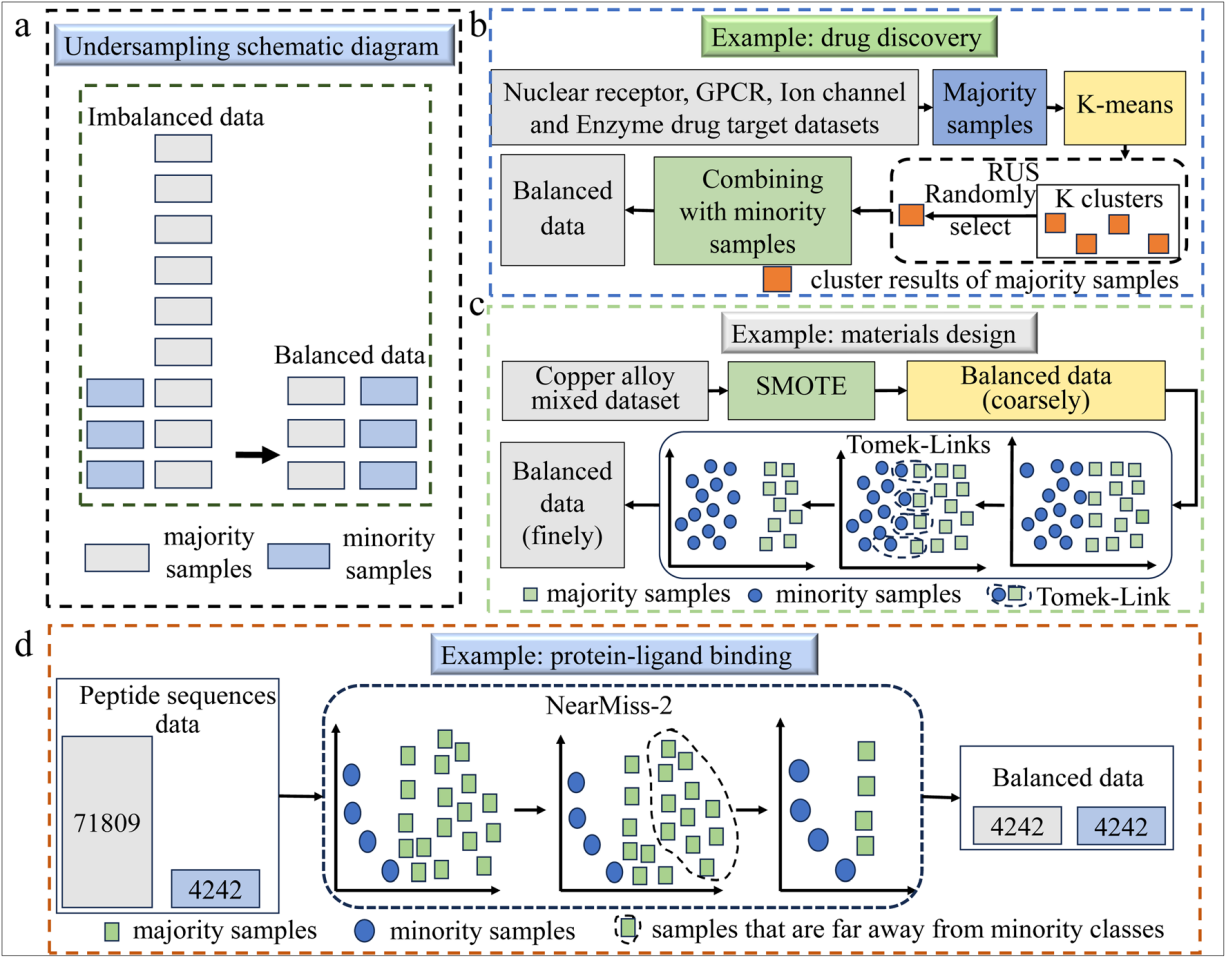
(a) A schematic diagram of undersampling method, demonstrating the approach of undersampling technique to balance the dataset. (b) This example demonstrates the application of a new method based on RUS technology in the realm of drug discovery.^25^ The majority samples in the drug target dataset are clustered using K-means clustering method and divided into different clusters. After that, the RUS method is used to randomly select a cluster from these clusters, repeat multiple times, and combine the selected cluster with minority samples in the original dataset to form a new balanced set. (c) This instance illustrates the use of the Tomek-Links approach for addressing imbalance in data within the realm of materials design.^26^ Initially, SMOTE is used to generate minority samples, making the dataset roughly balanced. Then, Tomek Links is used to identify and remove the majority samples in Tomek-Links (samples near the classification boundary) to clean the data, thereby refining the roughly balanced dataset into a finer one. (d) This example uses the NearMiss-2 method to address data imbalance within the domain of protein-ligand binding.^27^ Firstly, a training dataset of peptide sequences is constructed, containing 4242 minority samples with malonylation sites and 71 809 majority samples without malonylation sites. Next, the NearMiss-2 method is used to calculate the distance between each majority sample and each minority sample, and then the k farthest minority samples are selected to calculate the average distance to these k minority samples. Finally, the majority sample with the smallest average distance is retained to achieve data balance.

Undersampling is a data preprocessing technique that reduces the number of majority class samples to address class imbalance, enabling the model to focus more on minority class patterns. An illustration of undersampling is shown in Fig. 2a. By rebalancing the dataset, undersampling improves the model's predictive performance on minority classes. Commonly used undersampling methods include Random Under-Sampling (RUS),^28^ NearMiss,^27^ and Tomek Links.^29^

RUS randomly removes a portion of majority class samples to balance the dataset. The sampling rate is typically determined by the ratio of majority to minority class samples. After removing the excess majority samples, the resulting dataset is better balanced, allowing the model to learn from both classes in an unbiased manner. RUS has been successfully applied in various chemistry domains, including the prediction of anti-parasitic peptides,^28^ drug-target interaction (DTI) prediction,^30^ and compound–protein interaction prediction.^31^ In drug discovery,^25^ due to the greater number of non-interacting (negative samples) drug–target pairs than interacting (positive samples) drug–target pairs, this imbalanced dataset reduces prediction accuracy. Therefore, a new RUS-based method was used to process the data, as shown in Fig. 2b. While RUS is simple to implement and can reduce training time by decreasing the dataset size, it has potential drawbacks. Removing too many majority class samples can lead to the loss of important information, which may negatively affect model performance, particularly in drug discovery and genomics. In areas such as protein engineering and quantum chemistry, where intricate patterns and subtle variations in data are crucial, careful consideration is required to avoid discarding valuable information.

The NearMiss algorithm reduces the number of majority class samples while preserving key distribution characteristics, improving classifier performance, particularly in binary classification tasks. Its core principle is to select majority class samples that are closest to the minority class in the feature space for undersampling.

NearMiss is widely used due to its efficiency in handling high-dimensional data. Its robustness against noisy data and outliers, combined with scalability and ease of integration with other algorithms, makes it suitable for various applications in chemistry. For example, in 2022, Wang *et al.* applied the NearMiss-2 method to address imbalanced data in protein acetylation site prediction, significantly improving the Malsite-Deep model's accuracy in protein engineering.^27^ Their workflow is shown in Fig. 2d. Similarly, in molecular dynamics simulations, NearMiss is used to address data imbalance, which facilitates the identification of different conformational states of protein receptors.^32^

Despite its advantages, NearMiss may lead to the loss of valuable information due to undersampling, particularly in fields like drug discovery, catalyst design, and genomics. Additionally, due to its reliance on proximity in the feature space, NearMiss can struggle with capturing complex, nonlinear relationships, limiting its effectiveness in highly imbalanced or intricate datasets in protein–ligand binding or quantum chemistry.

The Tomek Links method reduces the number of majority class samples by identifying and removing those that are close to minority class samples in the feature space. This approach improves the model's ability to focus on the minority class by reducing class overlap. It works by identifying pairs of majority and minority class samples that are nearest neighbors, called Tomek Links, and removing the majority class samples from these pairs. This enhances the distinction between classes for model training.

Tomek Links has been applied in various chemical domains, including identifying glutarylation sites^29^ and pharmacophoric fragments of DYRK1A inhibitors,^33^ boosting the efficiency of experimental parameter optimization of nanometric solid solution alloys design^26^ (an illustration of the specific process is shown in Fig. 2c), and predicting compound–protein interactions.^34^ This method is particularly effective for noise reduction while preserving the overall data structure, improving model performance in fields like genomics, materials, and drug discovery. However, its reliance on identifying noise points based on proximity can risk removing valuable data. Additionally, its efficiency declines with larger datasets, which limits its applicability in certain large-scale contexts.

#### 2.1.3 Hybrid techniques

##### 2.1.3.1 SMOTE-Tomek links

The SMOTE-Tomek Links technique combines oversampling and undersampling to enhance dataset balance and classification performance.^35^ SMOTE synthesizes new minority class samples, while Tomek Links removes overlapping boundary samples, refining the dataset and reducing class overlap. This approach effectively mitigates data imbalance and overfitting, leading to clearer class boundaries and improved classifier accuracy and generalization. Widely applied in fields such as protein engineering,^29^ genomics, and transcriptomics,^36^ SMOTE-Tomek Links has demonstrated its ability to improve classification models, particularly for high-dimensional gene expression data, by facilitating the identification of key biomarkers. However, it can be computationally expensive, especially for large datasets, and excessive oversampling may still risk overfitting. Therefore, careful parameter tuning is essential to maximize the method's effectiveness, particularly in drug discovery and catalyst design.

##### 2.1.3.2 SMOTE-edited nearest neighbor (SMOTE-ENN)

SMOTE-ENN (edited nearest neighbor) is a hybrid resampling method by combining SMOTE's oversampling with the ENN technique to remove noisy majority class samples.^37^ This approach rebalances class distributions, enhancing the representativeness of minority classes while improving the model's robustness by reducing overfitting. SMOTE-ENN has been successfully applied in diverse chemical fields, such as protein–ligand binding^38^ and DTI prediction.^39^ Specifically, considering the large number of non-interaction class samples and the low proportion of interaction class samples in the DTI dataset, there is a significant class imbalance problem. Therefore, SMOTE-ENN technology was adopted to solve this problem, helping to improve the accuracy of drug–target interaction prediction.^40^ Although highly effective in improving model performance on imbalanced datasets, SMOTE-ENN is computationally intensive and sensitive to parameter selection. Careful parameter tuning is needed to mitigate risks such as generating poor-quality samples, particularly in noisy or unevenly distributed datasets.

#### 2.1.4 Cluster-based techniques

##### 2.1.4.1 Density-based spatial clustering of applications with noise-SMOTE (DBSCAN-SMOTE)

**Fig. 3:**
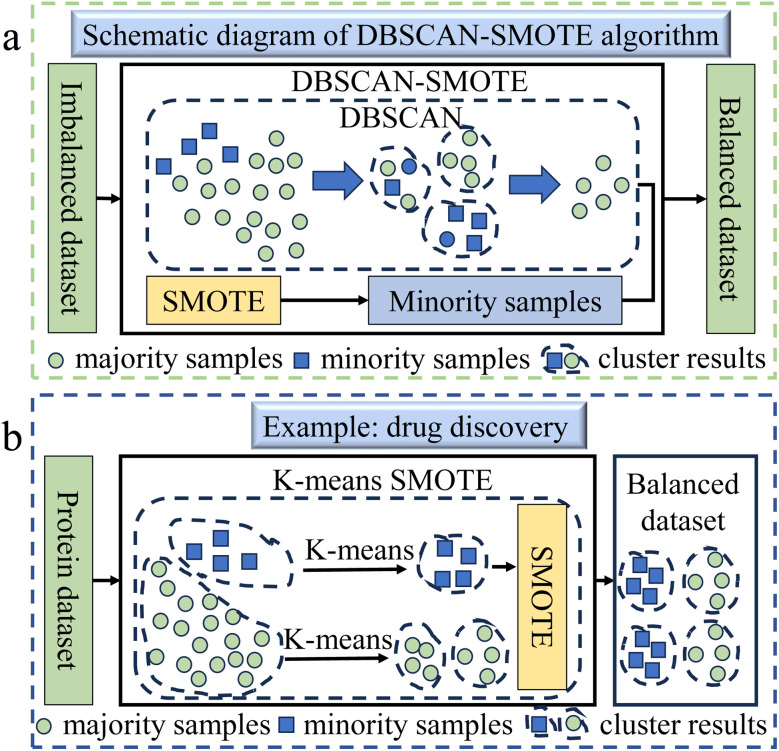
(a) The schematic diagram of DBSM algorithm flow.^41^ The process of DBSM includes two parts: undersampling and oversampling. For the undersampling part, apply DBSCAN to create clusters from all training sets. Then a portion of the majority samples is deleted from each cluster. The output of the undersampling technique is only majority samples. For the oversampling part, SMOTE is used to add synthetic sampless of minority samples to the training set. Therefore, the final output of the DBSM algorithm is a new training set consisting only of the majority samples from the undersampling part and the minority samples from the oversampling part. (b) This example demonstrates the application of the K-means SMOTE method in predicting bioluminescent proteins to address imbalanced data.^42^ Firstly, K-means is used to cluster the majority and minority samples separately to solve the problem of intra-class imbalance. Secondly, SMOTE is used for oversampling a small number of samples (luminescent proteins) to increase the number of minority samples and form a new balanced dataset with the majority samples.

DBSCAN-SMOTE (DBSM) is a hybrid method that combines the density-based clustering algorithm DBSCAN with SMOTE,^41^ as shown in Fig. 3a. In DBSM, DBSCAN identifies core, boundary, and noise points within clusters by using parameters like neighborhood radius and minimum sample size. SMOTE is then applied to the core points of these clusters, increasing the representation of minority class samples. This approach effectively reduces data imbalance and optimizes sample distribution, enhancing both the performance and generalization of classification models. DBSM is well-suited for imbalanced datasets with noise or irregular cluster shapes. It has broad applicability in chemistry. In predicting cervical cancer,^43^ Gowri and colleagues employed DBSCAN to tackle the data imbalance in cervical cancer datasets. The selection of DBSCAN was due to its capability to detect anomalous samples by examining the density of data points, obviating the requirement for predefined parameters. In the context of drug screening, Koh *et al.* used the DBSCAN method to process imbalanced data due to the fact that it can identify and classify different antagonists based on the density distribution of compound structures.^44^

DBSCAN-SMOTE handles noisy data and outliers well, making it effective in fields like genomics and protein–ligand binding, but its performance depends heavily on parameter selection and can be computationally expensive for large datasets.

##### 2.1.4.2 K-means SMOTE

K-means SMOTE is a hybrid technique created by integrating K-means clustering with SMOTE.^45^ It first partitions the data into clusters using K-means, then focuses on those with a higher proportion of minority class samples for targeted oversampling. Minority samples are generated between selected clusters to improve distribution, with sample density guiding the oversampling process. This approach enhances both the quantity and representativeness of minority class samples, improving model performance.

In the biomedical context, due to the diversity of disease subtypes or drug responses leading to uneven class distribution in the data, the K-means SMOTE method, by combining clustering and oversampling techniques, can effectively balance the imbalanced dataset while preserving its intrinsic structure, thereby enhancing the drug prediction model's ability to identify minority class samples.^46^ In the field of protein engineering, Nath *et al.* employed the K-means SMOTE method to manage imbalanced data, which is attributable to its capability to efficiently refine the class distribution in the dataset, catering to the challenge of low sequence similarity among bioluminescent proteins.^42^ An illustration of K-means SMOTE method is shown in Fig. 3b.

K-means SMOTE effectively improves model performance by generating realistic minority class samples, but its two-step process, consisting of clustering and oversampling, costs many computational resources and requires careful parameter optimization.

### 2.2 Data augmentation

#### 2.2.1 Noise addition

Gaussian noise addition is a widely used data augmentation technique in ML. By introducing controlled randomness based on the Gaussian distribution, it simulates real-world noise and variability in data, which forces models to focus on the essential and generalizable features of minority class samples, rather than overfitting to dominant patterns in the majority class. This helps counteract the issue of imbalance by making the model less sensitive to superficial trends in the data and better equipped to handle unpredictable environments.

This approach has proven effective in handling imbalanced data in various chemical applications. For example, in protein–ligand binding prediction, Lu *et al.* applied Gaussian noise addition to training data, since Gaussian noise can improve the model's adaptability to large-scale protein conformational changes and the ability of the model to identify hidden binding sites.^47^ Similarly, in drug discovery, Chakraborty *et al.* added Gaussian noise to the latent representation of autoencoders as the noise addition increased molecular diversity and complexity while maintaining the rationality of molecular structure.^48^

Gaussian noise addition improves model generalization on imbalanced data by simulating random disturbances, but its effectiveness depends on the careful tuning of noise levels to avoid distorting minority class features or compromising interpretability.

#### 2.2.2 Deep generative models

##### 2.2.2.1 Generative adversarial networks (GANs)

**Fig. 4:**
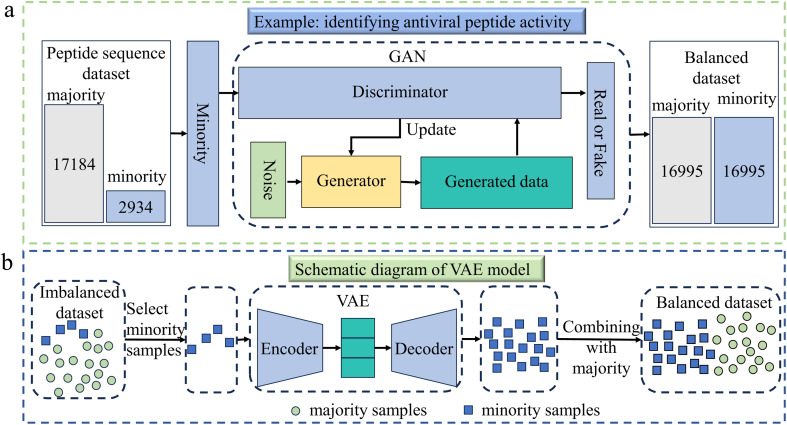
(a) This example demonstrates the application of generative adversarial network (GAN) in identifying antiviral peptide activity.^59^ Firstly, an imbalanced dataset was constructed, consisting of 2934 antiviral peptides (AVPs) and 17 184 non-antiviral peptides. The AVPs were used as input data to train the GAN model and then many AVP-like data were generated. Finally, the generated data were added to the original AVP data to achieve balance between the majority and minority samples. (b) The illustration of the variational autoencoder (VAE) algorithm for balancing data.^60^ It is divided into two parts: encoder and decoder. The former compresses the input into probabilistic latent representations, while the latter reconstructs data from latent space, the part between the encoder and the decoder. When applied to imbalanced data, VAE achieves balance between majority and minority classes by generating new samples for the minority class.

GANs, first proposed by Goodfellow *et al.* in 2014,^49^ are a class of DL models consisting of two neural networks: a generator and a discriminator. The generator's goal is to create realistic synthetic data, while the discriminator tries to distinguish between real and fake data. In the context of imbalanced data, GANs can be used to generate synthetic samples for the minority class. By training the generator to produce new, realistic samples of the underrepresented class, GANs help to increase the quantity and diversity of minority class data. This approach allows models to learn better representations of the minority class, reducing the bias towards the majority class. GANs have been successfully applied in many fields to handle class imbalance, such as drug design,^50–53^ materials design,^54^ protein engineering,^55,56^ catalyst design,^57^ and others. Due to the limitations of classical GANs in training stability and exploring certain regions of chemical space, Li *et al.* proposed a novel quantum GAN in 2021, which had a hybrid generator (QGAN-HG) for discovering small drug molecules.^58^ In predicting antiviral peptides, Lin *et al.* used GAN to address the issue of imbalanced antiviral peptide datasets,^59^ due to its ability to produce new samples that closely matched the distribution of real data. An illustration of balancing data process is given in Fig. 4a.

GANs effectively balance imbalanced data by generating diverse, high-quality minority class samples, but their unstable training and risk of mode collapse may limit their ability to fully capture minority class features.

##### 2.2.2.2 Variational autoencoders (VAEs)

A VAE is a type of generative model that learns to map input data to a continuous latent space, from which it can generate new data samples.^61,62^ It consists of two main components: an encoder that compresses the input into a probabilistic latent representation, and a decoder that reconstructs the data from the latent space. When applied to imbalanced data, VAEs can generate synthetic data by producing new samples for the minority class, as shown in Fig. 4b. The encoder–decoder structure of VAEs allows them to learn meaningful latent representations of the minority class, and by sampling from the latent space, new realistic data points can be generated. This helps mitigate the bias towards the majority class by enriching the diversity and quantity of minority class samples, making VAEs effective for handling class imbalance in fields such as drug discovery,^63,64^ protein engineering,^65^ molecular dynamics,^66^ and materials design.^67^

Recently, Schilter *et al.* used the VAE method in catalyst design to handle imbalanced data, based on the ability of VAE to autonomously learn meaningful structural features from the data. Compared to other existing advanced methods, VAE performed better in handling imbalanced datasets, as it not only maintained data distribution but also generated effective and innovative datasets.^68^ In protein–ligand binding, Ngo *et al.* used the VAE method, as it can learn information about the entire protein structure and utilize its powerful generative ability to generate ligands with high binding affinity and synthetic feasibility.^69^

VAEs effectively augment minority classes by generating new samples from a continuous latent space; however, their reliance on fixed distributions and tendency to produce blurry samples can constrain their ability to capture complex data features.

#### 2.2.3 Feature augmentation

Feature augmentation is a technique used in ML to create new features or modify existing ones by applying transformations, combinations, or domain-specific manipulations to the data. The goal is to enrich the feature space, enabling the model to learn more complex patterns and improve overall performance.^70–72^ Common methods of feature augmentation include polynomial features,^73^ feature interactions,^74^ mathematical features,^70,75^ and domain-specific transformations such as logarithmic scaling^76^ or statistical combinations.^77^

In imbalanced datasets, the minority class often lacks sufficient diversity, making it harder for the model to learn its patterns. By augmenting the features, new dimensions of variation can be introduced to the minority class, providing more informative and diverse data points. This allows the model to better distinguish the minority class from the majority class, reducing bias and improving classification accuracy. Feature augmentation works well when combined with other imbalance-handling techniques such as oversampling, enhancing the model's ability to generalize across both minority and majority classes and providing a more balanced representation of the data.

This method has been applied in many chemical fields, such as in DTI prediction^78^ and DDI prediction.^79^ In protein function prediction, Wan *et al.* proposed the FFPred-GAN method in 2020 and used feature augmentation to handle imbalanced data,^80^ which can effectively simulate the complex features of proteins in organisms without changing the distribution of the original data, while generating high-quality synthetic protein feature samples. Hayes *et al.* proposed the BTDT-MBO algorithm in 2024, which transformed molecular structures into informative feature vectors and employed a feature augmentation strategy, significantly improving the recognition capability of minority classes within molecular datasets.^75^ Additionally, in protein–ligand binding, Akbar *et al.* used feature augmentation techniques to handle imbalanced data owing to their capability to integrate information from multiple feature vectors and improve the model's recognition ability when parsing complex biological data.^81^

While feature augmentation can improve model performance on imbalanced data, it risks adding irrelevant features, overfitting, or noise, and does not directly address the core imbalance between majority and minority classes.

### 2.3 Algorithmic approaches

#### 2.3.1 Ensemble methods

##### 2.3.1.1 Boosting

**Fig. 5:**
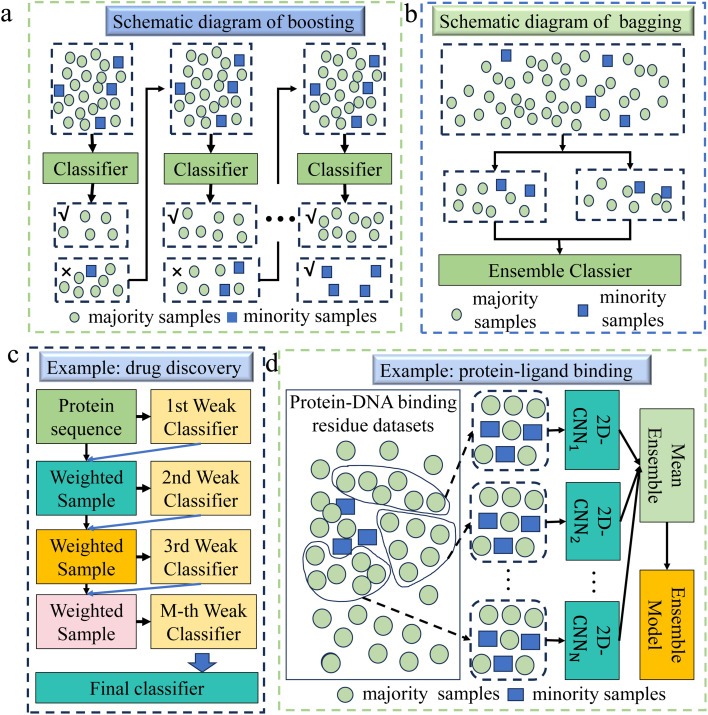
(a) A schematic diagram of the boosting algorithm. This method constructs a powerful classifier by connecting multiple weak classifiers. It uses an iterative process to make each subsequent classifier focus more on the misclassified minority class samples in the previous classifier's classification results, thus balancing the attention to minority and majority classes. (b) A schematic diagram of the bagging algorithm. It creates multiple subsets through random sampling and substitution, and it improves the recognition of minority classes by increasing the presence of minority samples in the subsets. (c) This example demonstrates the application of boosting in drug discovery.^83^ Firstly, an imbalanced dataset was constructed including proteins that can interact with drugs and proteins that cannot interact with drugs. The model then randomly selects samples with the same weight and chance from the dataset to train the first classifier model. Then, each classifier is tested on all samples in the dataset, and the weights of misclassified samples are updated iteratively to generate the final classification model from several individual weak classifiers. (d) This example demonstrates the application of bagging methods in the field of protein–ligand binding.^84^ Firstly, the majority samples and minority samples are separated from the original training set. Then, a certain number of samples are randomly selected from the majority samples and merged with the minority samples to form a new subset, which is repeated multiple times. Using the two-dimensional convolutional neural network (2D-CNN) framework to learn on each subset, an ensemble model is finally formed according to the mean ensemble strategy.

The boosting algorithm constructs a powerful model by concatenating multiple simple weak learners,^82^ as shown in Fig. 5a. It iteratively updates weights to make each subsequent learner focus more on misclassified minority class samples, thus balancing the attention to both minority and majority classes. Since the proposal of the boosting algorithm, many extensions have been proposed, such as Adaptive Boosting,^85^ Extreme Gradient Boosting (XGBoost),^86^ Gradient Boosting Decision Tree,^87^*etc.*

Boosting has many applications in chemical fields, such as drug discovery,^30^ catalyst design,^88^ protein engineering,^89^ protein–ligand binding,^90^ biomaterials design,^91^*etc.* Xue *et al.* selected the Gradient Boosting method for the design of biomaterials,^91^ due to its capability to effectively address data imbalance by incrementally building and refining models. This approach, in contrast to others, enabled a more precise identification of the intricate features influencing biomaterial properties. In genomics and transcriptomics, Liu *et al.* chose the XGBoost method to handle imbalanced data,^92^ thanks to its excellent generalization ability and higher prediction accuracy in handling high-dimensional problems, as well as its effectiveness in dealing with imbalanced data and categories. In drug discovery, Sikander *et al.* used the XGBoost method for accurate prediction of druggable proteins.^83^ This method demonstrated an excellent ability to handle high-dimensional data and strong resistance to overfitting, and its working principle diagram is shown in Fig. 5c.

Boosting enhances minority class classification by increasing sample weights, but its growing complexity with iterations requires careful tuning, and it often needs to be combined with techniques like data sampling or feature selection to improve efficiency.

##### 2.3.1.2 Bagging

Bagging is an ensemble method that creates multiple training subsets through random sampling with replacement, training independent models on each subset,^93^ as shown in Fig. 5b. The final output is obtained by aggregating predictions *via* voting or averaging. In imbalanced datasets, bagging can reduce bias by increasing the presence of minority class samples in some subsets, improving recognition of minority classes and reducing the influence of the majority class. While it stabilizes models like decision trees and mitigates overfitting, bagging alone does not well solve sample imbalance and often requires additional techniques like oversampling or undersampling for better minority class performance.

Bagging has a wide range of chemical applications, such as drug discovery,^94,95^ genomics and transcriptomics,^96^ catalyst design,^97^*etc.* In the study of drug toxicity detection,^98^ Gupta used an ensemble model based on bagging because the bagging method can effectively reduce the misjudgment of minority class samples by the model. Compared with a single classifier, it was more suitable for complex data classification in the field of biochemistry. Gong *et al.* employed the bagging method for addressing imbalanced data in druggable protein prediction due to its efficacy in mitigating model bias that arose from such imbalance. This method, in contrast to a single SVM classifier, offered a superior ability to integrate the significance of various features.^99^ In terms of protein–ligand binding, Hu *et al.* developed a method termed PredDBR to predict protein–DNA binding residues,^84^ as depicted in Fig. 5d, and employed the bagging method to address imbalanced data, since bagging was more adept at handling complex features in bioinformatics compared to a single model.

#### 2.3.2 Cost-sensitive learning

**Fig. 6:**
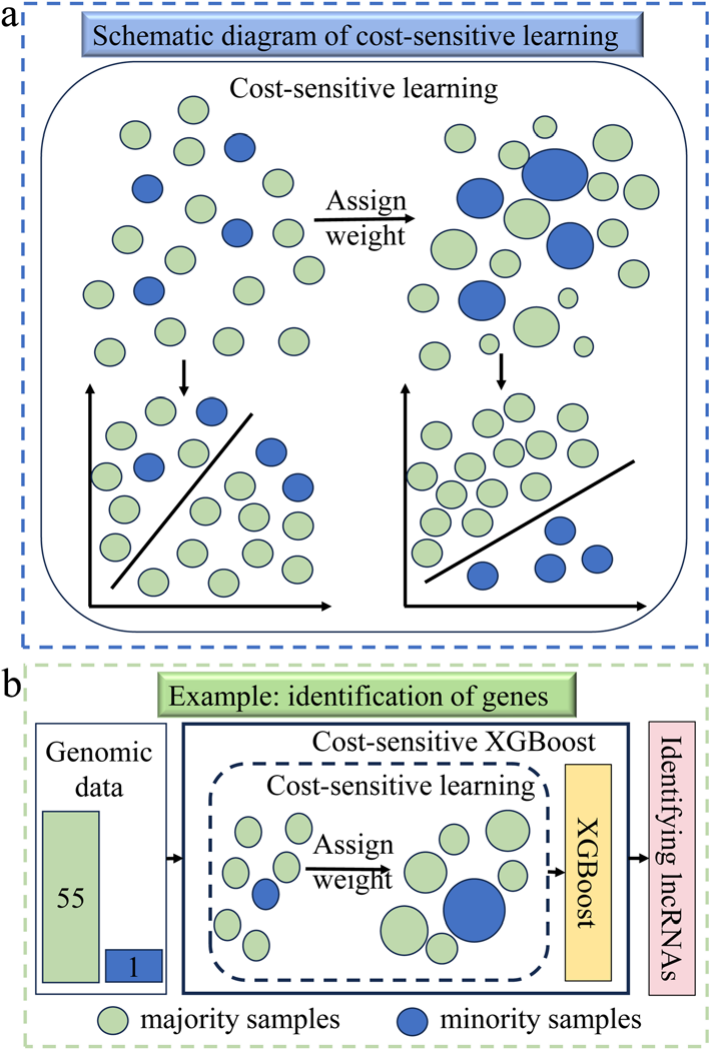
(a) A schematic diagram of the cost-sensitive learning (CSL) method. It assigns different weights to differently misclassified samples, focusing the model more on high-cost minority sample errors, thereby reducing the likelihood of misclassification. (b) This example demonstrates the application of the cost-sensitive XGBoost method in genomics and transcriptomics.^101^ The imbalanced genomic data (minority : majority = 1 : 55) is input into the cost-sensitive XGBoost framework for processing, using the CSL method to assign weights to the samples. Then, the XGBoost classifier is used for processing to obtain a balanced dataset for subsequent analysis or modeling processes.

Cost-sensitive learning (CSL) is an ML algorithm that evaluates the cost of different misclassified samples by applying different cost metrics, aiming to minimize the overall cost.^100^ It enables the model to pay more attention to high-cost minority sample errors by reweighting majority and minority samples, thereby reducing the probability of these errors and improving the performance on classification tasks in practical situations, as shown in Fig. 6a.

CSL has been widely applied in multiple fields of chemistry. For example, due to the highly imbalanced data in the DTI dataset, Aleb adopted a CSL method to improve model performance, which can assign higher weights to minority class samples in biological contexts, thereby more effectively identifying and predicting drug compound and protein interactions in drug design.^102^ In genomics and transcriptomics, Hazan *et al.* developed an advanced ML model called INFLAMeR for the identification of novel functional long non-coding RNAs (lncRNAs). This model employed a cost-sensitive XGBoost classifier to tackle the imbalance of training data, as depicted in Fig. 6b, with the rationale that the CSL allowed for the allocation of higher weights to minority categories, thereby enhancing the model's capability to detect key lncRNAs.^101^

CSL is efficient for large-scale molecular data and can addresses class imbalance by balancing performance across categories, improving predictive accuracy, especially in drug screening. However, improper cost settings may lead to overfitting minority classes and reduce generalization, and uncertainty in cost function design may impact model performance.

### 2.4 Feature engineering and selection strategies

Feature engineering is a key technology in data preprocessing, which involves extracting, processing, or creating new features from raw data to optimize the performance of ML models.^77^ It can not only help identify key features of minority classes, but also enhance the sensitivity of the model to minority class samples by designing specific features or transformations, improving the model's performance on imbalanced data.

Feature selection, as an important component of feature engineering, can remove redundant features and enhance the recognition accuracy of minority categories by filtering out the most relevant and important feature subsets in the dataset. Feature selection—including filter, wrapper, and embedded technologies as well as random feature selection—has various applications in fields such as drug discovery,^103^ protein engineering,^104–106^ genomics and transcriptomics,^107^*etc.*

#### 2.4.1 Filter technology

**Fig. 7:**
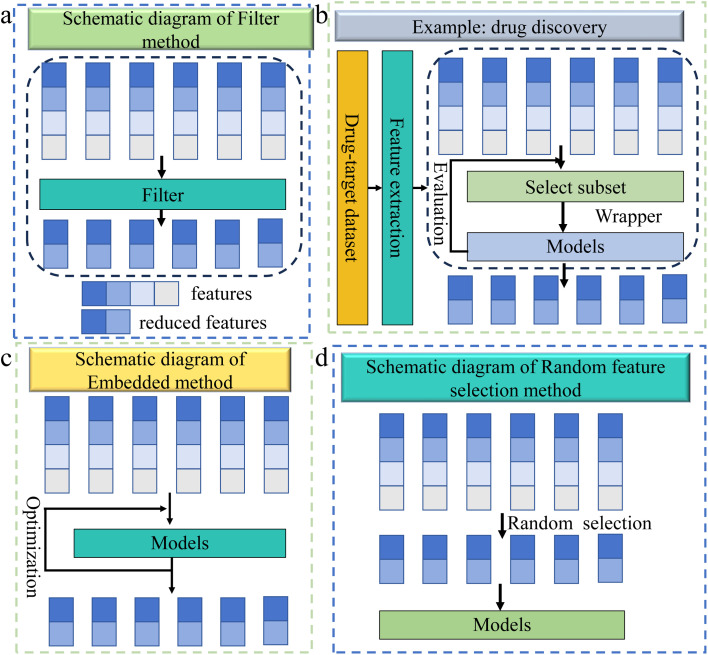
(a) The filter method sorts the six input samples (each with four features, different colors represent different features) directly based on different performance evaluation indicators and selects the feature with the highest score. (b) This example demonstrates the application of the wrapper feature selection method in the field of drug discovery.^108^ Firstly, through evaluating the extracted features, different weights are assigned for features. Then, a subset is selected from the feature set, and the wrapper method is used to choose the features that are most beneficial for model performance. (c) The schematic diagram of the embedded method, which combines feature selection with model training to ultimately obtain an optimal feature subset. (d) The workflow diagram of the random feature selection method, which randomly selects a subset of features from the entire feature set as the final feature subset.

Filter technology offers an efficient means for selecting subsets of pivotal features by quantifying the predictive power of each feature through the analysis of its statistical attributes or predefined criteria. The workflow for filter technology is shown in Fig. 7a. Filter technology can enhance a model's ability to recognize minority classes when dealing with imbalanced data, and its independence from models and high-dimensional data processing capabilities have enabled its application in multiple fields of chemistry.^109^

For instance, in 2023, Le *et al.* used the filter technology method for feature selection in protein engineering, both because the method can perform feature selection independently of the model and because it was more suitable for qualitatively evaluating feature importance in biomaterials.^110^ In the realm of catalyst design, Benavides-Hernández *et al.* opted for filter technology on the grounds that it successfully delineated the features with the most significant influence on catalyst efficacy while obviating the need for an augmented experimental workload.^111^

Filter technology has some limitations. It may not adequately capture the significance of certain features within a particular model or the intricate interactions among features. This technology may require further adjustments to ensure that the model does not lean towards the majority of class.

#### 2.4.2 Wrapper technology

The wrapper method can accurately identify key features that have significant predictive effects on minority categories, as it evaluates the effectiveness of features by repeatedly testing the interaction between feature subsets and the target model.

Case in point: in drug discovery, Mesrabadi *et al.* introduced a model for predicting DTI in 2023, using wrapper technology to remove irrelevant features. Compared to other methods, it was more suitable for predicting complex drug–target relationships in bioinformatics, ensuring that the selected features had a direct positive impact on model performance,^108^ as shown in Fig. 7b. In catalyst design, Shi *et al.* used wrapper technology to select the most critical subset for predicting adsorption energy from a large number of candidate features,^112^ as this technology ensured that the selected features contributed the most to the performance of the prediction model.

The wrapper method has high computational costs for large imbalanced datasets due to repeated model training, especially with few minority class samples, and is often combined with filtering or embedding techniques to optimize feature selection.

#### 2.4.3 Embedded technology

Embedded methods can automatically identify and select feature subsets that are crucial for minority class prediction during training, thereby reducing dependence on majority class features and enhancing sensitivity to minority classes. Its illustration is given in Fig. 7c.

The utility of embedded feature selection techniques spans various domains. For instance, in catalyst design, Ma *et al.* developed an ML-driven model to identify catalysts,^113^ which used embedded recursive feature elimination (RFE) to remove redundant features. A key benefit of this method is that it can dynamically select features during model training, enabling greater synchronization. Similarly, in drug discovery, Zhao *et al.* introduced a method based on convolutional neural networks (CNN) to embed relationship path features. Compared to other methods, embedded technology can better extract drug disease relationship path features.^114^

Although the embedded method can help the model better focus on minority categories, it may not fully reflect the importance of features or the complex interactions between features, which may require additional strategies to supplement when dealing with imbalanced data.

#### 2.4.4 Random feature selection

Random feature selection (RFS) reduces feature count without sacrificing accuracy and helps identify key features for minority classes in imbalanced datasets, improving prediction and reducing computational complexity while enhancing generalization. Its workflow is shown in Fig. 7d.

Recently, in DTI prediction, RFS was used in the training process of the model.^115^ In protein recognition research, Qiang *et al.* constructed a recognition model based on the RF model,^116^ where each tree was constructed based on a randomly selected subset of features. The use of random feature selection was to enhance feature representation, aiming to extract information from different perspectives of numerous feature descriptors and eliminate redundant and irrelevant features through the optimization of the feature space.

While RFS saves time and reduces overfitting, its randomness may cause accuracy fluctuations, especially in correlated chemical data, requiring optimization or combination with other methods for stable minority class predictions.

### 2.5 Evaluation metrics suitable for imbalanced datasets

When dealing with imbalanced data, traditional metrics in ML models like accuracy and precision are not suitable, as they can be skewed by the majority class, giving a false sense of high performance even when the minority class is poorly predicted. In contrast, balanced accuracy,^117^ the F1 score,^118^ Area Under the Receiver Operating Characteristic Curve (AUC-ROC),^119^ and Matthews correlation coefficient (MCC)^120^ are more suitable.

The F1 Score focuses on the minority class by addressing false positives and false negatives, making it useful when false negatives are costly. The mathematical formula for the F1 score is given as follows:1
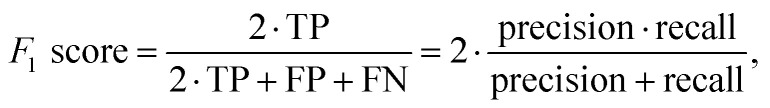
where actual positives that are correctly predicted as positives are called true positives (TP). Actual positives that are wrongly predicted as negatives are called false negatives (FN). Actual negatives that are correctly predicted as negatives are called true negatives (TN). Actual negatives that are wrongly predicted as positives are called false positives (FP).

The AUC-ROC curve evaluates a classifier's ability to distinguish between classes by measuring true positive and false positive rates across classification thresholds. The definitions of true positive and false positive rates are given by:2
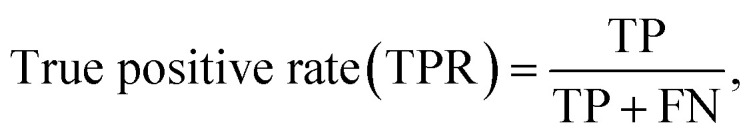
3
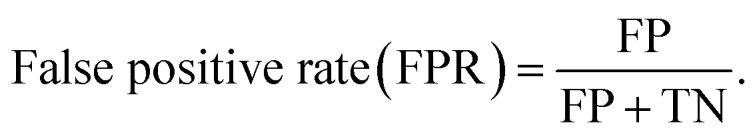


MCC offers a balanced evaluation, accounting for true/false positives and negatives and providing a comprehensive performance view even with imbalanced classes. The mathematical formula is given in the following form:4
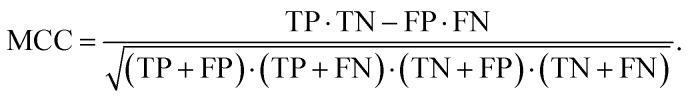


## 3 Perspectives for future directions and challenges

In reviewing techniques for addressing imbalanced data, we have analyzed their strengths, weaknesses, and applications in chemistry, as summarized in Table 1. In this chapter, we briefly give the rules of thumb for selecting suitable methods to address data imbalance issues, explore future research directions, offer unique perspectives, and discuss new approaches to better tackle the imbalanced data challenge in chemistry.

**Table 1:** An overview of major machine learning approaches and their strengths and weaknesses for data imbalance challenges

Methods		Strengths	Weaknesses	Ref
Resampling techniques	Oversampling techniques	Generate new minority class samples, while preserving the original feature distribution and mitigating overfitting	Introduce noisy data, struggle with complex decision boundaries, fail to account for internal distribution difference within the minority class, and high computational costs	6 and 7
	Undersampling techniques	Reduce training time by decreasing the dataset size, and robustness against noisy data and outliers	The loss of important information, and its efficiency declines with large datasets, which limits its applicability in certain large-scale contexts	28, 29 and 32
	Hybrid techniques	Synthesize new minority class samples and remove overlapping boundary samples, effectively mitigating data imbalance and overfitting	Computationally expensive for large datasets, and sensitive to parameter selection, particularly in noisy or unevenly distributed datasets	35, 37, 38 and 40
	Cluster-based techniques	Effectively reduce data imbalance and optimize sample distribution, enhancing both the performance and generalization of classification models	Depend on parameter optimization and can be computationally expensive for large datasets	41–45
Data augmentation	Noise addition	Make the model less sensitive to superficial trends in the data and better equipped to handle unpredictable environments	Need careful tuning of noise levels to avoid distorting minority class features or compromising interpretability	47 and 48
	Deep generative models	Learn better representations of the minority class, reducing the bias towards the majority class	Unstable training and risk of mode collapse, and reliance on fixed distributions and tendency to produce blurry samples	49, 50, 61, 62 and 68
	Feature augmentation	Provide more informative and diverse data points to better distinguish the minority class from the majority class, reducing bias and improving classification accuracy	Risk adding irrelevant features, overfitting, or noise, and does not directly address the core imbalance between majority and minority classes	70 and 70–75
Ensemble methods	Boosting	Adaptively emphasize misclassified minority instances, effectively reducing bias toward majority classes	The growing complexity with iterations requires careful tuning and often needs to be combined with techniques like data sampling or feature selection to improve efficiency	82–87
	Bagging	Reduce variance and improve stability by aggregating predictions from multiple independently trained models	Less effective at handling severe class imbalance due to reliance on random sampling, potentially neglecting minority classes	93–95
Cost-sensitive learning	Cost-sensitive learning	Enable the model to pay more attention to high-cost minority sample errors by reweighting majority and minority samples	Improper cost settings may lead to overfitting minority classes and reduce generalization, and uncertainty in cost function design may impact model performance	100 and 101
Feature engineering and selection strategies	Filter technology	Recognize minority classes with high-dimensional data processing capabilities	It may not adequately capture the significance of certain features within a particular model or the intricate interactions among features	109–111
	Wrapper technology	Accurately identify key features that have significant predictive effects on minority categories	High computational costs for large imbalanced datasets due to repeated model training	108 and 112
	Embedded technology	Automatically identify and select feature subsets that are crucial for minority class prediction during training	Feature selection may be biased toward majority class due to intrinsic optimization criteria	113 and 114
	Random feature selection	Simple, computationally inexpensive, and reduce variance by decorrelating selected features	Its randomness may overlook critical features vital for accurately predicting minority classes, leading to suboptimal classification performance	115 and 116

### 3.1 Rules of thumb for selecting suitable methods

Selecting suitable methods for addressing data imbalance issues requires the consideration of several key factors, including the severity of imbalance, dataset size, computational resources, and data complexity.

Firstly, for mild or moderate data imbalance cases (*e.g.*, minority-to-majority ratio is equal to or less than 1 : 10), simpler techniques such as oversampling or undersampling can be considered. For datasets with relatively small sizes and low dimensinality, oversampling like SMOTE can effectively enhance minority class representation without significantly increasing complexity. However, oversampling may inadvertently introduce noise or exacerbate overfitting, especially in cases where minority class samples are noisy or contain outliers. To mitigate this, combining oversampling with noise-filtering techniques or employing advanced variants such as Borderline-SMOTE is recommended.

Conversely, if the dataset is large, significantly imbalanced, or noisy, undersampling techniques often become the method of choice due to their computational efficiency. They streamline model training by reducing majority class samples, improving model responsiveness, and reducing resource consumption. However, undersampling should be performed carefully to avoid discarding valuable information. Techniques such as Tomek Links are recommended to selectively remove redundant majority class samples while preserving important data points.

In scenarios with severe imbalance (*e.g.*, minority-to-majority ratio of 1 : 50 or higher), traditional sampling methods alone may be insufficient. Leveraging more sophisticated approaches such as deep generative models like GANs becomes advantageous. Nevertheless, such methods require substantial computational resources and careful tuning, making them more suitable for projects where high accuracy and predictive performance justify the resource investment.

For datasets characterized by significant noise, feature redundancy, or high dimensionality, feature selection or augmentation methods should be employed. Cost-sensitive learning methods are particularly suitable for contexts where different misclassification costs are explicitly defined or can be quantified precisely. Adjusting misclassification costs allows models to prioritize correctly classifying minority class instances, directly addressing imbalance at the algorithmic level without additional sampling.

Finally, ensemble-based methods, such as Random Forest, AdaBoost, or gradient boosting like XGBoost, are consistently robust and effective. They inherently manage data imbalance by aggregating multiple weak classifiers, providing strong predictive accuracy and reduced variance. Combining ensemble methods with advanced sampling or feature-selection approaches can further enhance predictive performance, particularly in complex real-world applications.

### 3.2 Emerging trends in imbalanced data research in chemistry

In chemistry, recent research on the imbalanced data challenge reflects the convergence of interdisciplinary approaches and the development of novel methodologies.

#### 3.2.1 Multimodal data fusion

Recently, multimodal data fusion has gained attention for integrating data from different sources to provide a more comprehensive understanding. By combining molecular data, such as gene expression, protein–protein interaction,^121^ and DDI data,^122^ researchers can better explore structural features and interaction mechanisms. This approach enhances model learning capacity and performance, improving the detection of minority samples.

#### 3.2.2 Federated learning

Additionally, federated learning offers a potential avenue in handling imbalanced datasets by enabling collaborative model training without sharing raw data. This is particularly useful in drug discovery, where data from different laboratories, such as protein sequences^123^ and drug molecules,^124^ can be combined despite differences in size and diversity. Federated learning not only helps predict drug properties like activity and toxicity but also mitigates data imbalance, enhancing model accuracy and accelerating drug development.

#### 3.2.3 Self-supervised learning

Moreover, self-supervised learning has emerged as a burgeoning research direction. Unlike semi-supervised learning, it uncovers patterns directly from data without external labels. This allows it to process large volumes of unlabeled data, improving model performance on imbalanced datasets. In protein engineering, it could predict molecular properties^125^ and stability changes from mutations,^122^ helping to guide the synthesis of novel compounds and accelerate their development.

### 3.3 Physical-model-based data augmentation

One future research trend on data augmentation strategies is focusing on integrating physical models to generate virtual data with meaningful physical properties, thereby enriching datasets and improving model performance.

In this research field, molecular dynamics (MD) simulation plays a crucial role by simulating molecular motion and interactions, revealing conformations that experimental data may miss. This is especially relevant in areas like protein–ligand interactions^126^ and reaction mechanisms, where rare but important configurations are underrepresented. By generating such scenarios, MD could help build richer datasets, addressing data imbalance and improving models' ability to predict molecular properties and reactions.

Density functional theory (DFT) and molecular docking are pivotal in data augmentation strategies. DFT, a quantum mechanical approach, can generate critical data on chemical reactivity, catalytic activity, photophysical properties, nuclear magnetic resonance spectra,^127^ binding free energy,^128^ and molecular electronic structures,^129^ enhancing ML models' ability to predict molecular properties. Its broad applicability across molecular systems can help balance datasets and improve generalization. Through predicting interactions between molecules and receptors,^130^ molecular docking could provide binding patterns and affinity data. Both techniques can enrich datasets, especially for imbalanced data, improving the accuracy of models in predicting molecular interactions and properties.

The integration of physical models with ML is emerging as a key trend. By incorporating physical laws into ML frameworks, models can enhance prediction accuracy and interpretability. Advances in computational power enable physics-based data augmentation, creating more diverse training datasets and fostering more generalizable chemical models. For example, thermodynamic and statistical mechanics simulations can generate data on equilibrium constants, free energy changes, and reaction rates, enriching datasets, addressing imbalances, and boosting model performance.

### 3.4 Large-language-model-based data augmentation

As chemistry faces the challenge of imbalanced datasets, the rise of deep learning (DL), especially Large Language Models (LLMs), such as ChatGPT and Gemini, offers promising solutions. LLMs excel in data augmentation, as demonstrated by Sarker *et al.*, who showed that models like ChatGPT improve accuracy in drug identification and classification.^131^ Furthermore, models like Chemformer, introduced by Ross Irwin in 2022,^132^ can handle a range of chemical tasks, including sequence-to-sequence and discriminative tasks, showcasing the potential of LLMs in chemical research.

LLMs could offer innovative solutions for addressing imbalanced chemical data by learning from diverse representations, such as textual descriptions and chemical structures. For example, generating molecular structures from textual representations demonstrates how LLMs can enrich datasets and mitigate the data imbalance challenge, advancing chemical data analysis.^133^

### 3.5 Mathematics-enabled data augmentation

A trending topic in data science is the integration of AI with advanced mathematics, such as differential geometry, algebraic topology, combinatorics, geometric topology, *etc.*^70,71^ Recently, mathematical AI has become an emerging paradigm in molecular data sciences, including drug design competitions,^134,135^ the discovery of viral evolution mechanisms,^136^ the forecasting of emerging dominant variants,^137,138^ protein engineering,^139^ protein mutation prediction,^140^ toxicity prediction,^141^ drug addiction analysis,^142^ polymer property,^143^*etc.* The multiscale topology-enabled transformer by Chen *et al.* gives rise to the best prediction of protein–ligand binding affinities.^144^ This mathematical approach captures stereochemistry,^145^ which is missing in typical sequence-based molecular language models. As such, mathematics-enabled generative models will be a new approach for data augmentation.

Additionally, the graph-based Merriman–Bence–Osher method has been utilized to handle imbalanced data.^75^ It leverages its diffusion process to propagate label information across the graph, enabling better representation of minority classes in semi-supervised learning tasks.

### 3.6 New materials design and manufacture

While the experimental synthesis of the minority class offers the ultimate solution to imbalanced data, balanced datasets play a key role in AI-assisted active design and manufacture of new materials. Enhanced computational power and refined algorithms are accelerating breakthroughs in new materials development by ensuring more equitable data distribution.

Balancing datasets significantly improves the accuracy of ML models in predicting new materials. Recent studies using techniques like feature engineering^146^ have led to more reliable models that excel in predicting material properties and optimizing synthesis pathways, accelerating material research and development. The use of balanced datasets is driving the integration of high-throughput experimentation and computational simulations in material innovation.^147^ By reducing bias and improving data efficiency, these datasets guide experimental design and, when combined with simulations, enable researchers to explore a wider range of parameters, accelerating the discovery and development of new materials.

Balanced datasets are crucial for fostering material diversity. By creating and utilizing these datasets, researchers can more effectively explore existing material databases, uncovering unique materials and driving innovative material design.

### 3.7 Persistent challenges and areas for further investigation

In chemical research, the management of imbalanced data continues to encounter significant challenges. Although various solutions have been proposed in recent years, several fundamental issues remain unresolved and demand urgent attention. First, the combination of small data^148^ and imbalanced data poses one of the most significant challenges in molecular science. Data scarcity and imbalance are especially pronounced across numerous chemical applications. Due to the inherent difficulty in producing large volumes of balanced and high-quality data during experiments, particularly when studying new materials or rare compounds, or when performing toxicity evaluations, finding efficient methods to collect, share, and integrate data has become a pressing concern.

Additionally, the absence of standardized processes for data handling and dissemination between different research projects and laboratories further complicates the resolution of imbalanced data issues. The current mechanisms for data sharing require substantial improvement. Although a growing number of academic institutions and journals advocate for open data practices, operational challenges persist. For example, inconsistent data repository formats and incomplete or insufficiently detailed data documentation hinder effective reuse. Thus, establishing a standardized platform, spearheaded by relevant organizations or funding bodies, to regulate data submission and validation processes is crucial for enhancing data transparency and quality.

While several approaches have been applied to mitigate the imbalanced data challenge,^2,149^ the resilience and generalizability of these methods still require significant refinement. Many existing techniques struggle when applied to small sample sizes,^148^ making it difficult to address the complex molecular structures and reaction pathways inherent to chemical research. Consequently, future studies should prioritize the development of more precise and field-specific data augmentation and modeling techniques. Notably, enhancement methods based on physical models may hold considerable promise in rectifying data imbalance, although these approaches are still in the nascent stages of exploration.

To drive progress in this area, forthcoming research should aim to create robust frameworks that facilitate the widespread implementation of imbalanced data technologies. Moreover, researchers should emphasize the thorough validation of new algorithms, particularly through testing across various chemical application scenarios, to ensure their practical utility and consistency. These efforts will provide a strong foundation and theoretical backing for addressing the imbalanced data problem in the chemical sciences.

## Data availability

No primary research results, software or code have been included and no new data were generated or analysed as part of this review.

## Author contributions

CZ contributed to writing the Section 2 and the preparation of figures; LK contributed to writing the Section 3; JJ contributed to writing the remainder and the preparation of figures and table; NH, YZ, HQ, BZ, TZ, and GW revised the manuscript.

## Conflicts of interest

There are no conflicts to declare.
